# Hyperactivation of mTORC1 by an endogenous *raga-1* gain-of-function mutation does not reduce lifespan in *C.&nbsp;elegans*

**DOI:** 10.17912/micropub.biology.001520

**Published:** 2025-04-25

**Authors:** Tatiana M. Moreno, Michelle E. Brown, Caroline Kumsta

**Affiliations:** 1 Graduate School of Biomedical Sciences, Sanford Burnham Prebys Medical Discovery Institute, La Jolla, California, United States; 2 Development, Aging and Regeneration Program, Sanford Burnham Prebys Medical Discovery Institute, La Jolla, California, United States

## Abstract

Inhibition of mTORC1, a conserved nutrient-sensing complex, extends lifespan across model organisms, but the effects of mTORC1 hyperactivation are less understood. RagA, a GTPase essential for mTORC1 activation, can be locked in its active GTP-bound state through gain-of-function mutations, such as Q63L in *C.* *elegans* RAGA-1. We found that transgenic expression of *raga-1[Q63L]* mutation ( *egIs12* ) decreases lifespan without hyperactivating mTORC1, suggesting mTORC1-independent effects or transgene toxicity. In contrast, we show that a CRISPR-generated Q63L mutation at the endogenous *raga-1* locus ( *viz128)* hyperactivates mTORC1 without affecting lifespan, challenging the paradigm that mTORC1 hyperactivation accelerates aging. Thus, genetic context and potential compensatory mechanisms may contribute to mTORC1-mediated lifespan regulation, at least in metazoans.

**Figure 1. Endogenous raga-1 gain-of-function mutation ( viz128 ) increases phospho-RSKS-1 without affecting lifespan in C. elegans:**
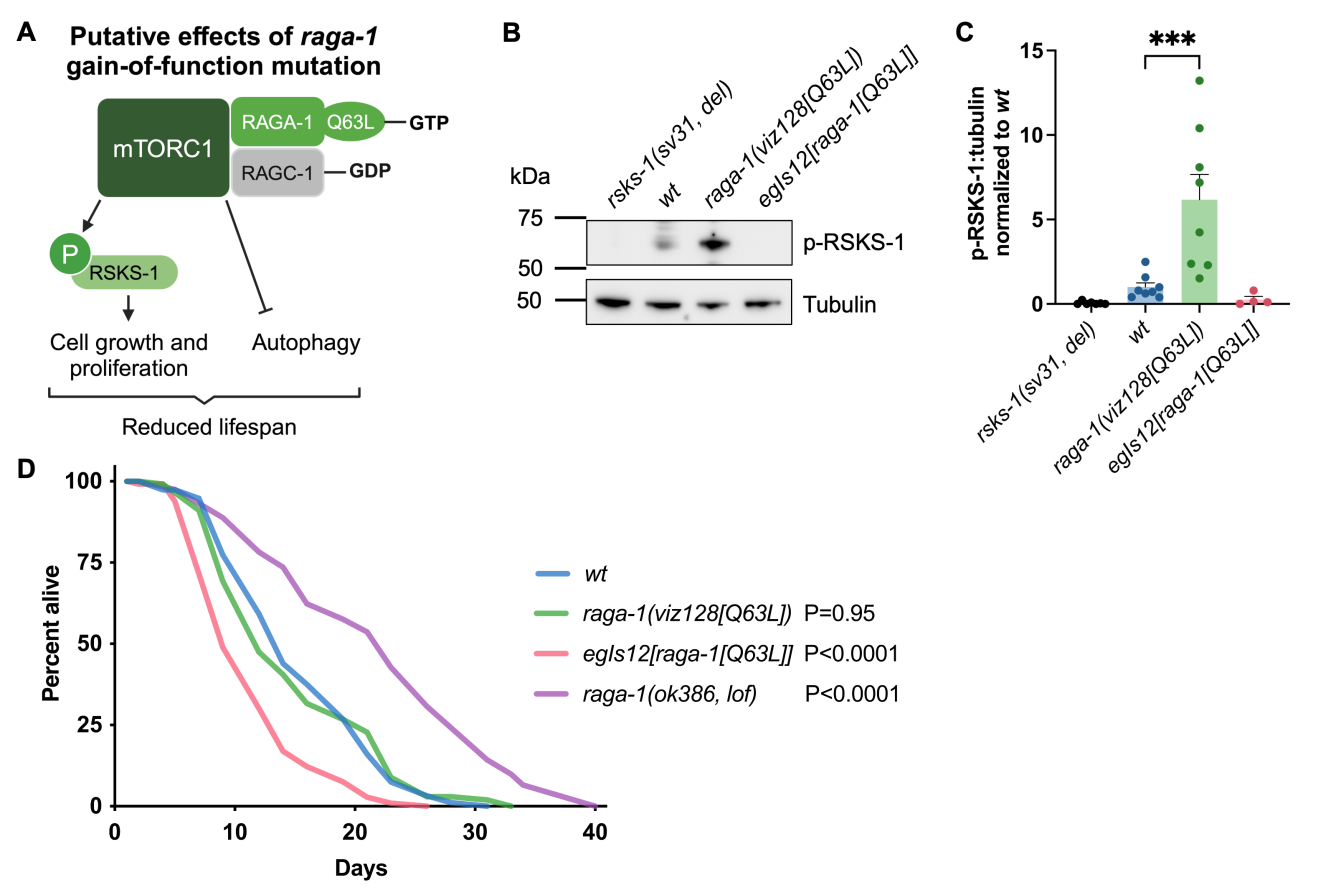
**(A)** Model of the putative effects of Q63L gain-of-function mutation in *C. elegans* RAGA-1 (RagA[Q66L] mutation in humans). RAGA-1[Q63L] is expected to lead to constitutive GTP binding, stabilizing its active conformation in a dimer with GDP-bound RAGC-1, hyperactivating mTORC1, and leading to phosphorylation of downstream targets, including RSKS-1/S6K to stimulate cell growth and others to inhibit autophagy, contributing to lifespan shortening. **(B)** Representative Western blot and **(C)** quantification of phospho-RSKS-1(T404), conserved from human phospho-p70-S6 Kinase(T389), in day-1 old *rsks-1(sv31)* , wild type ( *wt* ), *raga-1(viz128[Q63L])* , and *egIs12[raga-1[Q63L]]* animals. *raga-1(viz128[Q63L])* and *egIs12[raga-1[Q63L]]* were outcrossed four times to wild type, see **Reagents** . *rsks-1(sv31, del)* carries a 2,673 bp deletion including T404, and is used as a negative control. n=4-8. ***P<0.001 by mixed-effects analysis with Holm-Šídák's correction. No other comparisons to *wt* were significant. **(D)** Representative lifespan analysis of *wt* , *raga-1[Q63L]* and *raga-1* loss-of-function mutants. *raga-1(ok386, lof)* carries a 1,242 bp deletion spanning from 11 bp upstream of the Q63 codon through the 3' UTR. *wt* (mean lifespan (MLS): 15.5 days, N=98/116) compared with *raga-1(viz128[Q63L])* (MLS: 15.0 days, N=103/112), *egIs12[raga-1[Q63L]]* (MLS: 11.3 days, N=108/117), and *raga-1(ok386, lof)* (MLS: 21.9 days, N=101/120). Statistical comparisons by log-rank test. See **Extended Data Table 1** for additional lifespan experiments.

## Description

The mechanistic target of rapamycin complex 1 (mTORC1) is a conserved protein complex that regulates cellular growth and metabolism in response to environmental and nutritional cues. Under nutrient-rich conditions, mTORC1 becomes activated and promotes anabolic processes such as cell growth and proliferation while simultaneously inhibiting catabolic pathways such as autophagy. Conversely, during nutrient scarcity or stress, mTORC1 activity is reduced, leading to the activation of autophagy to recycle cellular components and maintain energy homeostasis (Saxton and Sabatini 2017; Papadopoli et al. 2019).

Inhibition of mTORC1, either through genetic means or pharmacological compounds including rapamycin, reproducibly extends lifespan and can delay several hallmarks of aging including dysregulated nutrient sensing, disabled macroautophagy, and loss of proteostasis (Mannick and Lamming 2023; Papadopoli et al. 2019). These effects are observed across diverse model systems, including in *C. elegans* (Vellai et al. 2003; Hansen et al. 2007; Robida-Stubbs et al. 2012; Zhang et al. 2024), *Drosophila* (Kapahi et al. 2004; Bjedov et al. 2010) and mice (Harrison et al. 2009; Wu et al. 2013). In contrast, increased mTORC1 activity has been observed in several human and rodent tissues with age (Markofski et al. 2015; Joseph et al. 2019; Tramutola et al. 2015), and mTORC1 activation via gain-of-function mutations of the heterodimeric Ras-related small GTP-binding proteins (Rag GTPases), which positively regulate mTORC1 complex formation and activity, is associated with shortened lifespan in mammalian models (de la Calle Arregui et al. 2021; Ortega-Molina et al. 2024). However, the characterization of models hyperactivating mTORC1 to directly study its effects on aging and related pathways is still limited, particularly in genetically tractable systems, such as *C. elegans* . Understanding the physiological effects of mTORC1 hyperactivation is critical to uncover how mTORC1 contributes to aging and age-related pathologies.

A key regulatory mechanism of mTORC1 involves the Rag GTPases (Sancak et al. 2008; Kim et al. 2008), which act as nutrient sensors that facilitate mTORC1 localization to lysosomes, critical for its activation. In mammals, RagA/B and RagC/D form obligate heterodimers, while *C. elegans* only has one homolog of each family RAGA-1 and RAGC-1 . Under nutrient-rich conditions, GTP-bound RagA/B and GDP-bound RagC/D form an active heterodimer that interacts directly with mTORC1 to activate downstream targets (Sambri et al. 2024). These include ribosomal S6 kinase (S6K), which promotes protein synthesis (Brown et al. 1995), and Unc-51-like kinase 1 (ULK1), which inhibits autophagy initiation (Kim et al. 2011). Upon low-nutrient conditions, the GTP-bound state of RagA/B is reduced, leading to mTORC1 inactivation, and dephosphorylation of mTORC1 targets (Sambri et al. 2024). Through this regulation, the Rag GTPases mediate a dynamic switch between anabolic and catabolic processes, coupling mTORC1 activity to nutrient availability.

While these regulatory principles are well-characterized in mammals, their exact roles in regulating mTORC1 in *C. elegans* remain less understood. Evidence suggests that RAGA-1 and RAGC-1 act as positive regulators of mTORC1 in *C. elegans* , as loss-of-function mutations phenocopy reduced mTORC1 activity in mammalian systems, leading to increased autophagy, decreased protein synthesis, and extended lifespan (Robida-Stubbs et al. 2012). However, the developmental defects caused by loss of *raga-1* or *ragc-1* are less severe than those observed with core mTORC1 components (Fukuyama et al. 2012), indicating that mTORC1 may retain some activity independent of RAGA-1 and RAGC-1 (Blackwell et al. 2019; Schreiber et al. 2010). Gain-of-function mutations, such as *raga-1 [Q63L]* , putatively lock RAGA-1 in its active GTP-bound state, and provide a model for mTORC1 hyperactivation ( **Fig. 1A** ). The transgenic *C. elegans* strain *egIs12 [ raga-1 p:: raga-1 [Q63L]+ofm-1p::gfp]* overexpresses the *raga-1 [Q63L]* mutation, alongside wild-type *raga-1* , and exhibits a drastically shortened lifespan (Schreiber et al. 2010; Gerisch et al. 2020; Huang et al. 2022). However, neither mTORC1 activity nor RAGA-1 expression levels have been directly assessed in this strain, leaving it unclear whether the reported lifespan reduction is due to mTORC1 hyperactivation or alternative pathways influenced by RAGA-1 activation.

To further investigate the role of mTORC1 hyperactivation in *C. elegans* aging, we analyzed the effects of the *raga-1 [Q63L]* mutation on mTORC1 activity and lifespan. Our study included the previously characterized transgenic *egIs12 [ raga-1 [Q63L]]* strain (Schreiber et al. 2010), and a newly CRISPR-generated strain, *raga-1 ( viz128 [Q63L])* (Trimmer and Arur 2021), which carries the *raga-1 [Q63L]* mutation at its endogenous gene locus. The *raga-1 ( viz128 [Q63L])* strain allowed us to study the effects of RAGA-1 hyperactivation without additional transgenic expression, providing a more direct model of mTORC1 hyperactivation. We assessed mTORC1 activity by measuring phosphorylation of mTORC1 target RSKS-1 (T404), the *C.&nbsp;elegans* ortholog of mammalian S6K(T389) ( **Fig. 1A** ). While *rsks-1 ( sv31 )* mutants, which carry a large deletion including T404, showed no p-RSKS(T404) signal, p- RSKS-1 (T404) levels were significantly increased in the *raga-1 ( viz128 [Q63L])* mutant, but not in the *egIs12 [ raga-1 [Q63L]]-* carrying strain, indicating that only the endogenous mutation is sufficient to increase mTORC1 activity ( **Fig. 1B, C** ). To further explore the functional consequences of altered mTORC1 activity via *raga-1* modulation, we assessed lifespan in the *raga-1 [Q63L]* strains. As a control for the effects of reduced mTORC1 activity on lifespan, we included a *raga-1 ( ok386 )* loss-of-function mutant, *raga-1 ( ok386 , lof)* ( **Fig. 1D** ), which significantly extended lifespan, as previously shown (Schreiber et al. 2010). The *egIs12 [ raga-1 [Q63L]]* strain exhibited a markedly reduced lifespan compared to wild type ( **Fig. 1D** ), consistent with previous reports linking transgenic *raga-1 [Q63L]* expression to accelerated aging (Schreiber et al. 2010; Gerisch et al. 2020; Huang et al. 2022). In contrast, the *raga-1 ( viz128 [Q63L])* strain, despite increased mTORC1 activity, surprisingly displayed a normal lifespan comparable to wild type ( **Fig. 1D** ).

Our findings reveal distinct molecular changes and lifespan phenotypes in strains carrying the *raga-1 [Q63L]* mutation, highlighting that RAGA-1 and mTORC1 activity measured via RSKS-1 phosphorylation, and aging in *C. elegans* are not fully interdependent, and further investigation is needed to elucidate the coupled and independent functions of each pathway component.

The *raga-1 ( viz128 [Q63L])* strain exhibited increased p- RSKS-1 (T404) levels, indicating elevated mTORC1 activity, yet showed no significant difference in lifespan compared to wild type. This finding is inconsistent with the prevailing paradigm that mTORC1 hyperactivation universally accelerates aging, and suggests that mTORC1 hyperactivation, at least to this degree, may not be sufficient to impact lifespan in metazoans. It remains unclear whether other mTORC1 targets are similarly phosphorylated in this mutant, warranting further investigation to map the broader effects of this *raga-1* gain-of function mutation on mTORC1 activity. Further characterization of mTORC1-associated phenotypes, such as autophagy, nucleolar size, and lipid storage, could help identify which mTORC1-dependent pathways are selectively activated, inhibited or unaffected in the context of endogenous *raga-1 [Q63L]* mutation. These phenotypes could help explain why lifespan remains unchanged in the *raga-1 ( viz128 [Q63L])* mutants, despite increased RSKS-1 phosphorylation.

In contrast, the *egIs12 [ raga-1 [Q63L]]* strain exhibited decreased levels of p- RSKS-1 (T404) compared to wild type (0.26-fold, corresponding to a 73.7% reduction), although this difference did not reach statistical significance (P=0.67, mixed-effects model). This strain also displayed a markedly shortened lifespan. The combination of these two observations suggests again that mTORC1 activity, RSKS-1 phosphorylation, and longevity are not strictly coupled. Several non-exclusive explanations could account for this. First, the shortened lifespan could result from *raga-1 [Q63L]* overexpression toxicity effects, independent of mTORC1 signaling. This explanation is consistent with the shortened lifespan but does not readily account for the reduced p- RSKS-1 levels. To test whether the reduced lifespan in *egIs12 [ raga-1 [Q63L]]* is independent of RSKS-1 , lifespan of long-lived *rsks-1* mutants (Hansen et al. 2007) expressing *egIs12 [ raga-1 [Q63L]]* could be assessed; if *egIs12 [ raga-1 [Q63L]]* shortens *rsks-1* mutant lifespan, it would support the hypothesis that overexpression toxicity, rather than mTORC1 signaling via RSKS-1 , drives the phenotype. Second, the residual wild-type *raga-1* expression could mitigate the hyperactivating effects of the mutant allele, thereby limiting mTORC1 activation and RSKS-1 phosphorylation; however, this would more likely normalize p- RSKS-1 levels and lifespan rather than reducing both. Assessing lifespan in *raga-1 ( viz128 [Q63L])/+* heterozygotes, which, like *egIs12* , would express both wild-type and the *raga-1* gain-of-function mutation *,* could help distinguish between dosage-dependent effects and transgene-specific toxicity. If such heterozygotes exhibit normal lifespan, this would argue against a simple buffering model and instead support a contribution of transgene overexpression to the *egIs12* phenotype. Third, a dominant-negative effect could explain the reduced p- RSKS-1 levels. A dominant-negative effect occurs when a mutant protein interferes with the normal function of the wild-type protein, often by forming non-functional complexes that block downstream activity. If overexpressed mutant RAGA-1 [Q63L] bound and sequestered endogenous RAGC-1 into non-productive heterodimers, this could suppress mTORC1 activation. While this would be consistent with the reduction in RSKS-1 phosphorylation, an extended lifespan rather than a shortened lifespan would be expected, and a further inhibition of mTORC1 (e.g., via *let-363* RNAi) should fail to extend lifespan, which could be tested in future experiments. Finally, It is also possible that the reduced lifespan observed in *egIs12 [ raga-1 [Q63L]]* is driven by tissue-specific effects of mTORC1 hyperactivation that do not translate into a detectable global increase in RSKS-1 phosphorylation by Western blot. A recent study demonstrated that neuron-specific expression of *raga-1* in a long-lived *raga-1* loss-of-function mutant is sufficient to abolish its longevity phenotype (Zhang et al. 2019), highlighting the potential for distinct outcomes depending on the tissues in which mTORC1 signaling is activated.

Our study has certain limitations, such as using tubulin for Western blot normalization rather than total levels of RSKS-1 /S6K due to the lack of a robust RSKS-1 /S6K antibody validated in *C. elegans* . Although RSKS-1 /S6K phosphorylation may not fully reflect all functional outputs of mTORC1 activity, it remains the most comparable readout to mammalian studies available. Additionally, the *raga-1 ( viz128 [Q63L])* strain showed some variability in p- RSKS-1 levels, which may reflect genetic drift, as this variability became more pronounced when the strain was cultured over several weeks. To ensure data reliability, we recommend using recently-thawed populations and multiple biological replicates when using this strain or assessing RSKS-1 phosphorylation in any experimental context.

Overall, these findings challenge the current paradigm that mTORC1 hyperactivation universally reduces lifespan. We demonstrate that mTORC1 hyperactivation, as implied by increased p- RSKS-1 /S6K levels, in *C. elegans raga-1 ( viz128 [Q63L])* mutants, is not sufficient to accelerate aging. These results underscore the complexity of mTORC1 signaling in aging, highlighting how the degree and mode of hyperactivation influence its biological outcomes and necessitates further research into its context-specific effects.

## Methods

*C. elegans* cultivation

*C. elegans* were maintained at 20ºC on NGM plates, fed with *Escherichia coli* OP50 , and cultured following standard protocols (Brenner 1974). AUM1693 ( *raga-1 ( viz128 ))* and BZ1290 ( *egIs12 [ raga-1 [Q63L]; ofm-1p::gfp]* ) were outcrossed 4 times to N2 wild type to generate KUM91 and KUM142 , respectively. VC222 ( *raga-1 ( ok386 )* ) and VB633 ( *rsks-1 ( sv31 )* ) were not outcrossed. *raga-1* mutant genotypes were confirmed by PCR and Sanger sequencing. The *C. elegans* strains used in this study are listed under **Reagents** .

Western blots

Age-synchronized animals (N=100) were hand-picked on day 1 of adulthood into Eppendorf tubes containing 30 µL M9 buffer (0.6% sodium phosphate dibasic, 0.3% potassium dihydrogen phosphate, 0.5% sodium chloride, 0.025% magnesium sulfate heptahydrate) using a platinum wire, and immediately washed 3-5 times with additional M9 buffer to remove all OP50 bacteria. After the final wash, M9 buffer volume was adjusted to ~10 µL. 2 µL 6X Laemmli buffer (Thermo Scientific, #J61337.AC) was added, and samples were flash frozen in liquid N _2_ . Worms were lysed by two rounds of freeze-thaw in liquid N _2_ and briefly spun down in a tabletop centrifuge after each thaw, then boiled at 95ºC for 10 min. Samples were loaded onto a 4-12% Bis-Tris protein gel (NuPAGE) in MOPS running buffer (NuPAGE, #NP0001) and proteins were separated using 110 V. Proteins were transferred to a PVDF membrane (Millipore, #IPVH85R) using a Novex Mini Cell system (Invitrogen, #EI0001) at 30 V for 1.5 hours on ice. Membranes were blocked with 5% milk in Tris-buffered saline containing 0.05% Tween-20 (TBS-T) for 30 minutes, followed by immunoblotting using primary anti-phospho-p70 S6 Kinase (Thr389) (Mak et al. 2020) (1:1,000 in 1% milk in TBS-T; overnight incubation at 4ºC) and secondary anti-mouse IgG HRP (1:10,000 in 1% milk in TBS-T) (see **Reagents** ). Western blots were developed using SuperSignal chemiluminescence reagent (Thermo Scientific, #34577 or #34095) and imaged on a ChemiDoc imager (Bio-Rad Laboratories, Inc.). To normalize protein loading, blots were stripped for 4 min using stripping buffer (Thermo Scientific, #46430), blocked again with 5% milk in TBS-T, probed with primary anti-tubulin (1:1,000 in 1% milk in TBS-T; overnight incubation at 4ºC) and secondary anti-rabbit IgG HRP (1:10,000 in 1% milk in TBS-T), and developed and imaged as described above. Protein expression was quantified using Image Lab 6.1.0 build 7 (Bio-Rad Laboratories, Inc.). Statistical analysis was performed using PRISM 10.4.0 software (GraphPad) and P-values were calculated using a mixed-effects model with Holm-Šídák's correction.

Lifespan

Lifespan was assayed at 20ºC as previously described (Hansen et al. 2005). A total of 108-140 age-synchronized worms were placed on six to seven 6 cm NGM plates seeded with OP50 bacteria at the L4 larval stage (lifespan day 0). Worms were transferred away from progeny to new 6 cm NGM plates seeded with OP50 bacteria until post-reproductive, after which worms were scored three times per week until dead. Worms were scored as dead when no movement was observed upon gentle prodding with a platinum wire. Worms that experienced internal hatching, desiccated on the edge of the plate, escaped, or were accidentally killed were censored. Statistical analysis was performed using OASIS 2.0 at https://sbi.postech.ac.kr/oasis2/ (Han et al. 2016), and P-values were calculated with the log-rank (Mantel–Cox) method. See **Extended Data Table 1** for a summary of all lifespan experiments.

## Reagents

**Table:** 

***C. elegans* strain **	**Genotype**	**Source**
**N2**	*Wild type*	Kumsta Lab, originated from Hansen Lab
**KUM91**	*raga-1 ( viz128 [Q63L]) II *	AUM1693 (Trimmer and Arur 2021) 4X outcrossed to N2
**KUM142**	*egIs12 [ raga-1 p:: raga-1 [Q63L]; ofm-1p::gfp] *	BZ1290 (Schreiber et al. 2010) 4X outcrossed to N2
**VC222**	*raga-1 ( ok386 ) II *	CGC
**VB633**	*rsks-1 ( sv31 ) III *	(Hansen et al. 2007)

**Table:** 

**Antibody**	**Animal and clonality**	**Source**
**Anti-Phospho-p70 S6 Kinase (Thr389)**	Mouse monoclonal	Cell Signaling catalog #9206, lot #30
**Anti-α-Tubulin**	Rabbit polyclonal	Cell Signaling catalog #2144, lot #7
**Anti-mouse IgG, HRP-conjugated**	Horse	Cell Signaling catalog #7076, lot #38
**Anti-rabbit IgG, HRP-conjugated**	Goat	Cell Signaling catalog #7074, lot #33

## Data Availability

Description: Extended Data Table 1: Lifespan analysis of wild-type and raga-1 mutant worms.. Resource Type: Dataset. DOI: https://doi.org/10.22002/kgd2h-f8s29
